# Impact of psychologically tailored hand hygiene interventions on nosocomial infections with multidrug-resistant organisms: results of the cluster-randomized controlled trial PSYGIENE

**DOI:** 10.1186/s13756-019-0507-5

**Published:** 2019-03-25

**Authors:** Thomas von Lengerke, Ella Ebadi, Bettina Schock, Christian Krauth, Karin Lange, Jona T. Stahmeyer, Iris F. Chaberny

**Affiliations:** 1 Hannover Medical School, Centre for Public Health and Healthcare, Department of Medical Psychology, Carl-Neuberg-Str. 1, OE 5430, 30625 Hannover, Germany; 2 Hannover Medical School, Centre for Laboratory Medicine, Institute of Medical Microbiology and Hospital Epidemiology, Carl-Neuberg-Str. 1, OE 5214, 30625 Hannover, Germany; 3 Leipzig University Hospital – AöR, Department for Diagnostics, Institute of Hygiene, Hospital Epidemiology and Environmental Medicine, Johannisallee 34, 04103 Leipzig, Germany; 4 Hannover Medical School, Centre for Public Health and Healthcare, Institute of Epidemiology, Social Medicine and Health Systems Research, Carl-Neuberg-Str. 1, OE 5410, 30625 Hannover, Germany

**Keywords:** Hand hygiene compliance, Nosocomial infections, Multidrug-resistant organisms, Psychological tailoring, Intensive care units, Physicians and nursing staff

## Abstract

**Background:**

Professional hand hygiene compliance represents a multifaceted behaviour with various determinants. Thus, it has been proposed to apply psychological frameworks of behaviour change to its promotion. However, randomized controlled trials of such approaches, which also assess nosocomial infections (NIs), are rare. This study analyses data of the PSYGIENE-trial (PSYchological optimized hand hyGIENE promotion), which has shown improvements in compliance after interventions tailored based on the Health Action Process Approach (HAPA), on rates of NIs with multidrug-resistant organisms (MDROs).

**Methods:**

A parallel-group cluster-randomized controlled trial was conducted on all 10 intensive care units and two hematopoietic stem cell transplantation units at Hannover Medical School, a German tertiary care hospital. Educational training sessions for physicians and nurses (individual-level intervention) and feedback discussions with clinical managers and head nurses (cluster-level) were implemented in 2013. In the “Tailoring”-arm (*n* = 6 wards), interventions were tailored based on HAPA-components, which were empirically assessed and addressed by behaviour change techniques. As active controls, *n* = 6 wards received untailored educational sessions of the local “Clean Care is Safer Care”-campaign (Aktion Saubere Hände: “ASH”-arm). From 2013 to 2015 compliance was assessed by observation following the World Health Organization, while alcohol-based hand rub usage (AHRU) and NIs with multidrug-resistant gram-negative bacteria, Methicillin-resistant *Staphylococcus aureus* or Vancomycin-resistant *Enterococcus* were assessed following national surveillance protocols. Data were analysed at cluster-level.

**Results:**

In the “Tailoring”-arm, interventions led to a decrease of 0.497 MDRO-infections per 1000 inpatient days from 2013 to 2015 (*p* = 0.015). This trend was not found in the “ASH”-arm (− 0 . 022 infections; *p* = 0.899). These patterns corresponded inversely to the trends in compliance but not in AHRU.

**Conclusions:**

While interventions tailored based on the HAPA-model did not lead to a significantly lower incidence rate of MDRO-infections compared to control wards, a significant reduction, compared to baseline, was found in the second follow-up year in the “Tailoring”- but not the "ASH"-arm. This indicates that HAPA-tailored hand hygiene interventions may contribute to the prevention of NIs with MDRO. Further research should focus on addressing compliance by interventions tailored not only to wards, but also leaders, teams, and individuals.

**Trial registration:**

German Clinical Trials Register/International Clinical Trials Registry Platform, DRKS00010960. Registered 19 August 2016-Retrospectively registered, https://www.drks.de/drks_web/navigate.do?navigationId=trial.HTML&TRIAL_ID=DRKS00010960. http://apps.who.int/trialsearch/Trial2.aspx?TrialID=DRKS00010960.

**Electronic supplementary material:**

The online version of this article (10.1186/s13756-019-0507-5) contains supplementary material, which is available to authorized users.

## Introduction

Noncompliance with hand hygiene guidelines remains a universal challenge [1, 2]. At the same time the most recent Cochrane review on interventions to improve hand hygiene compliance in patient care, while stating that existing interventions can potentially lead to some improvements, could draw no conclusion about which interventions lead to clinically significant increases in compliance and reduced nosocomial infection (NI) rates [3]. Since professional hand hygiene compliance represents “… a complex behaviour with numerous motivators and barriers” [4] p. 203, it has been proposed to apply psychological frameworks of behaviour change to its promotion [4, 5]. However, randomized controlled trials of such psychological approaches are rare, especially regarding studies which include NIs as outcomes. This is evident in the systematic review on psychological strategies to improve professional hand hygiene by Srigley and colleagues [4], which found one cluster-randomized controlled trial (C-RCT) not reporting NIs, and three studies examining NIs but using weak experimental designs. Also, these studies lacked “… clear descriptions indicating how interventions were designed to address theoretical behavioural constructs” [4] p. 207. This is particular problematic since an earlier review on behaviour change determinants associated with hand hygiene promotion strategies by Huis and colleagues showed that the strategies’ effectiveness was highly correlated with the number of determinants addressed [6].

One C-RCT which was not included in the review by Srigley and colleagues [4] but clearly described its addressed determinants was the HELPING HANDS-trial conducted among nurses in three Dutch hospitals [7, 8]. It evaluated a team- and leaders-directed strategy, and achieved an increase in compliance from 20 to 53% after six months, while in control wards receiving education, reminders, feedback, and optimized materials and facilities only, an increase from 23 to 46% was observed [7]. The difference could be partly explained by social influences (team and leadership support) [8]. However, a major limitation was that NI-rates were not empirically assessed in the participating wards but based on estimates from the literature [9]. Furthermore, by design the trial did not highlight person-centred approaches, e.g. goal setting and reward incentives, experiential methods, and psychological processes that determine behaviour change sustainability and habitualization [4, 6, 10–12]. Thus, level ≥ 2-evidence [13] in terms of (cluster-)randomized controlled trials of psychological approaches to reduce NIs by increasing hand hygiene compliance is still needed. This would follow the call for stronger study designs in NI-prevention [3, 14].

One such study is the PSYGIENE-trial (PSYchological optimized hand hyGIENE promotion) at Hannover Medical School, a tertiary care university hospital in Lower Saxony, Germany, which has used a two-arm parallel-group pre−/post-test C-RCT design on all of the centre’s 10 intensive care units (ICUs) and two hematopoietic stem cell transplantation units [15]. The trial examined whether educational training sessions and feedback discussions psychologically tailored on the basis of behavioural constructs from the “Health Action Process Approach (HAPA)” [15–20] were more effective in improving hand hygiene of physicians and nurses and in reducing NIs than the respective interventions of the local “Clean Hands-Campaign” (“Aktion Saubere Hände”, ASH). The ASH-campaign is an adaptation of the multi-modal “Clean Care is Safer Care”-programme for healthcare institutions by the World Health Organization (WHO) [5]. As implementation strategies, it endorses high-level administrative support, participation in one introductory and yearly courses to exchange experiences and learn about current developments in NI prevention, the introduction of WHO’s “My Five Moments of Hand Hygiene”-approach, continuing education, high availability of alcohol-based hand rub dispensers, measurement of alcohol-based hand rub usage (AHRU), and regular participation in a national hand hygiene day and in network workshops [21–23]. Both the participation in the campaign and the assessment of compliance by observation are voluntary. The campaign has developed promotional and reminder materials, an e-learning tool, a film, standard educational lectures, compliance observation tools, and a surveillance system for AHRU to facilitate hand hygiene promotion in the participating healthcare institutions. Infection control personnel and members of the administration are trained to achieve safety climate change. In addition, the ASH provides materials based on the WHO framework assessment tool for implementation evaluation [24] at regular intervals.

After initial successes in terms of hand hygiene improvements, a relapse in compliance had been observed in the ICUs and hematopoietic stem cell transplantation units at Hannover Medical School. Specifically, the compliance rate had increased from 56% in the year 2008 (baseline) to 66% in 2011, but dropped again to 55% in 2013 (which happened to be the final year of funding of the national campaign by the German Ministry of Health) [25]. Thus, a need for action was perceived to test improved interventions against those used in the local ASH, especially regarding sustainability.

In PSYGIENE, the HAPA was chosen as the trial’s theoretical compliance model for two reasons. First, despite serving as the national campaign’s official compliance model, it had neither been appropriately recognized nor adopted by the local ASH-team at that time. Second, the HAPA describes compliance on a staged continuum from developing motivation for a given behaviour to planning, implementation and routinisation of the behaviour. Thus, like the Transtheoretical Model of Change [26, 27] it qualifies as a stage theory of behaviour, allowing those applying it to differentiate healthcare workers in terms of their hand hygiene experience and behavioural stage [4, 10]. In other words, it enables to maximise the “‘appropriateness’ of determinants” [6], p.10 addressed by interventions to the healthcare workers in any given specific context.

As behavioural determinants, the HAPA specifies risk perception, outcome expectancies, self-efficacy expectancies, goal intentions, action and coping planning, action control, and external resources and barriers (both perceived and actual). As the HAPA has so far not been widely used and described within the field of infection prevention and control, Additional file 1: Table S1 gives an overview of its key constructs (see Additional file). The main differences to the Theory of Planned Behaviour, being the psychological framework most often used in hand hygiene studies so far [4], are threefold. First, the HAPA includes perceptions of risk, which seems essential when applying theories of compliance to healthcare workers since they are sensitised to concepts such as risks and probabilities as part of a system in which more than 95% of studies are quantitative [28]. Notably, this is independent of the level of numeracy which healthcare workers actually achieve [29].

Second, the Theory of Planned Behaviour presupposes a direct link between the intention to comply and the behaviour itself, which has been shown to be moderate for self-reported and practically absent for observed professional hand hygiene [30, 31]. In contrast, the HAPA bridges this gap between intention and behaviour by including the concept of implementation intentions. These include individual plans of when, where and how to behave (action planning) and to deal with relapses (coping planning).

Third, the HAPA includes action control, a self-regulative strategy where the ongoing behaviour is evaluated continuously (and, at best, automatically) against behavioural standards (e.g. knowledge of guidelines). The two latter concepts particularly allow to conceptualise hand hygiene not only as a deliberate behaviour, and a problem of poor motivation, but also as a habit [4, 10, 14].

So far, effects of HAPA-tailored interventions used in the PSYGIENE-trial on hand hygiene compliance assessed by direct observation have been reported [15]. Following the recent Cochrane review [32], tailored interventions were defined as planned measures to improve professional practice which take account of prospectively identified determinants of practice. While the wards randomized into the two study arms did not differ in baseline compliance in 2013, the tailored interventions, which used behaviour change techniques [33] deliberately selected to address the described behavioural constructs as assessed empirically on the participating wards (see below, Methods), led to increased compliance in 2014 and in 2015. In contrast, compliance in the “ASH”-arm initially increased but dropped again in 2015. Thus, tailored interventions appeared to lead to more sustainable compliance increases. Following up on these results, the present paper analyses how NIs with multidrug-resistant organisms (MDRO) developed in the two trial arms. MDRO-infections were prioritised because earlier studies in the German healthcare context have shown an increasing MDRO prevalence on ICUs from 2013 to 2015 (0.34 to 0.39 per 1000 patient days) [34], and significantly more adverse health outcomes and higher costs of NIs with resistant pathogens, regardless of whether they were compared with overall or drug-susceptible controls [35–37]. Besides, results on AHRU as a possible surrogate parameter for hand hygiene compliance are reported for the first time. In particular, AHRU is included because its measurement represents a substantial element of national hand hygiene campaigns not only in Germany [38], but other countries such as France [39], and England and Wales [40], as well.

## Methods

The trial’s methods, including study design and assessment of hand hygiene compliance rates, have been detailed earlier [15]. Thus, they are summarised here and appended by methods related to the assessments of AHRU and MDRO-infections.

### Study design

A single-centre C-RCT on Hannover Medical School’s 10 ICUs and two hematopoietic stem cell transplantation units as clusters was conducted. Overall, *n* = 515 physicians and *n* = 572 nurses worked on these wards at baseline (beginning of the year 2013). Six wards received tailored educational training sessions and feedback discussions (“Tailoring”-arm):

- *n* = 3 surgical ICUs (neurosurgery; cardiothoracic, transplantation and vascular surgery; general, abdominal and transplant surgery),
- *n* = 1 medical ICU (respiratory medicine),
- *n* = 1 interdisciplinary ICU (anaesthesiology), and
- *n* = 1 paediatric ICU (pulmonology and neonatology).

Six other wards received ASH-educational training sessions (“ASH”-arm):

- *n* = 2 surgical ICUs (plastic, hand and reconstructive surgery; trauma surgery),
- *n* = 1 medical ICU (cardiology and angiology),
- *n* = 2 hematopoietic stem cell transplantation units (paediatric haematology and oncology; hematology, hemostasis, oncology and stem cell transplantation), and
- *n* = 1 paediatric ICU (cardiology and internal medicine).

Table 1(a) describes the two study arms in terms of adult patients’ age, and mortality rates, across the three study years. While adult patients were about one to one and a half years older in the “ASH-”study arm (with larger variability in this arm), mortality rates were approximately two percent higher in the “Tailoring”-arm. Table 1(b) presents the number of employed physicians and nurses in the year 2013, i.e. at the beginning of the trial, and participation rates in the educational training sessions, which varied from 44.1 to 54%. Regarding feedback discussions in the “Tailoring”-arm, all *n* = 6 clinical managers and *n* = 6 head nurses received this intervention (not shown in Table 1; see *Interventions* below).

**Table 1:** a) Age of and mortality among adult patients across the three study years, and b) physicians’ and nurses’ participation rates in educational training sessions in the intervention year 2013, by study arms

	a)		b)			
	Mean age of adult patients (standard deviation)*	Mortality rate (95% confidence interval)	Physicians working in study arm in the intervention year 2013	Physicians participating in at least one educational training session in the intervention year 2013	Nurses working in study arm in the intervention year 2013	Nurses participating in at least one educational training session in the intervention year 2013
“Tailoring”-study arm						
2013	60.6 years (3.4 years)	7.3% (6.7–8.0%)	*n* = 315	*n* = 139 (participation rate: 44.1%)	*n* = 367	*n* = 190 (participation rate: 51.8%)
2014	61.0 years (3.9 years)	7.0% (6.4–7.6%)				
2013	61.8 years(4.1 years)	7.5% (6.8–8.2%)				
“ASH”- study arm						
2013	62.1 years(8.0 years)	5.2% (4.4–6.0%)	*n* = 200	*n* = 108 (participation rate: 54.0%)	*n* = 205	*n* = 94 (participation rate: 45.9%)
2014	62.1 years(8.2 years)	4.8% (4.1–5.6%)				
2013	62.8 years(8.9 years)	5.8% (5.0–6.7%)				

*Weighted means and standard deviations

### Participants

*Selection criteria:* None other than employment in a study ward as physician or nurse.

*Setting:* Hannover Medical School is a tertiary care university hospital specializing in surgery. In 2014, 60,173 fully inpatient treatment cases were recorded. Overall, it has a total of 1459 beds, and operates five surgical, two internal medicine, and two paediatric ICUs, one interdisciplinary ICU, and two hematopoietic stem cell transplantation units (total beds: 178).

### Interventions

Educational training sessions for physicians and nurses (individual-level intervention) and feedback discussions with clinical managers and head nurses of the participating wards (cluster-level) were implemented in 2013. In the “Tailoring”-arm, these interventions were tailored on the basis of empirically assessed psychological determinants of hand hygiene compliance. These determinants were identified using data obtained in a survey between November 26, 2012 and January 25, 2013 on the wards involved in the trial (response rates: physicians 71%, nurses 63%). The survey questionnaire, which was developed by the researchers [15, 17, 18] following HAPA-recommendations [16], assessed the compliance determinants that represent the key psychological factors according to the HAPA, i.e., risk perceptions, outcome expectancies, self-efficacy expectancies, intentions, action and coping planning, action control, and ward-specific resources and barriers (see Additional file 1: Table S1). In addition, key informant interviews based on principles of problem-centred interviewing [41] regarding typical daily routines/activities were conducted with the responsible ward physicians and head nurses (March 19 to May 7, 2013; response rates: 100%). In the tailoring arm in 2013, appropriate behaviour change techniques were selected for the training sessions and the feedback discussions. This selection, which was based on the expertise of the medical psychologists involved in the PSYGIENE-trial and performed in coordination with the leading Hospital Epidemiology Department and the health economists involved, defined the tailoring. A total of 29 behaviour change techniques were used in the tailoring arm of the trial. Fifteen techniques were used in the ASH arm (the interventional strategies used in the ASH arm have also been described, for comparison). Additional file 2: Table S2 lists those behaviour change techniques which were used in both arms, and those used in the “Tailoring”-arm only (see Additional File; for more details, see [15]). Other interventions to prevent and control different types of NIs (e.g. urinary tract, bloodstream, surgical site, and respiratory tract infections) such as device use, availability of hand rub dispensers, promotional and reminder material, antibiotic stewardship, and daily antiseptic body washings with octenidine did not differ between study arms (see, however, the limitations-section in the Discussion below).

### Outcomes

*Hand disinfection.* Compliance rates were assessed via direct observation of employees’ hand hygiene following WHO’s gold standard [42] and calculated for each year based on summated observations per trial arm. All observers were members of the Hospital Epidemiology Department; thus, neither clinical managers nor head nurses nor physicians or nurses on the wards were involved in this task. Cluster-level AHRU was recorded in accordance with the national ASH-protocol and expressed in millilitres (ml) per patient day [43].

*MDRO-infections.* Nosocomial infections with multidrug-resistant gram-negative bacteria (MRGN), methicillin-resistant *Staphylococcus aureus* (MRSA) and vancomycin-resistant *Enterococcus* (VRE) were assessed by German National Nosocomial Infection Surveillance System-standards [44] (and thus definitions of the Centers of Disease Control and Prevention [45]), and calculated as incidence densities (infections per 1000 inpatient days) for both trial arms for each of the three study period years. In this context, as VRE all relevant pathogens are counted by the surveillance system, i.e. *Enterococcus spp.* including *E. faecium, E. faecalis, E. gallinarum* and *E. casseliflavus*. A corresponding assertion holds for MRGN, except that Extended Spectrum Beta-Lactamase (ESBL) producing bacteria are not considered [46].

### Randomization

The 10 ICUs and two hematopoietic stem cell transplantation units were divided in two groups of six according to their mean hand hygiene compliance rates in 2008–2012 (median split: 63%). Cluster randomization was performed within these groups so that 3 units in each group were randomized to each trial arm. This aimed at harmonizing the compliance levels in the 2 trial arms before the start of the PSYGIENE-trial.

### Blinding

While the wards and the assessors of NI outcomes and AHRU were blinded, staff implementing interventions, observing hand hygiene compliance, and analysing data were not.

### Statistical methods

Data were analysed at cluster-level. Differences in MDRO-infection incidence densities and AHRU were analysed using the general linear model repeated measures-procedure in IBM® SPSS® Statistics 25 with “trial arm” as between-wards factor. Contrasts between study years and their interactions with the “trial arm”-factor are reported. For compliance differences OpenEpi 3.03a had been used to determine confidence intervals and significance of differences between study years across trial arms (Breslow-Day tests with chi-square tests) [47]. Because hand hygiene opportunities in trial arms did not generate differing sampling errors, no weighted analyses were performed. Statistical significance was set at *p* < 0.05 (two-tailed).

## Results

### Overview of MDRO-infections during the study period

Overall, 92 MDRO-infections were recorded during the study period. These included 13 MRSA-, 39 VRE- and 40 MRGN-infections. Additional file 3: Table S3 gives an overview of which MDROs occurred in the two study arms per year (see Additional File). Regarding VRE, exclusively NIs with *E. faecium* occurred on the participating wards during the study. In total, these infections represented the majority of MDRO-infections in the “Tailoring”-arm (33 of 64), whereas this pertained to MRGN-infections in the “ASH”-arm (18 of 28). In both arms, MRSA was the MDRO causing the lowest number of NIs. Regarding MRGN, gram-negative MDROs resistant to three of four classes of antimicrobials (3MRGN) were more frequent that those resistant to all four classes (4MRGN) [48].

### Effects on NI incidence rates

**Fig. 1:**
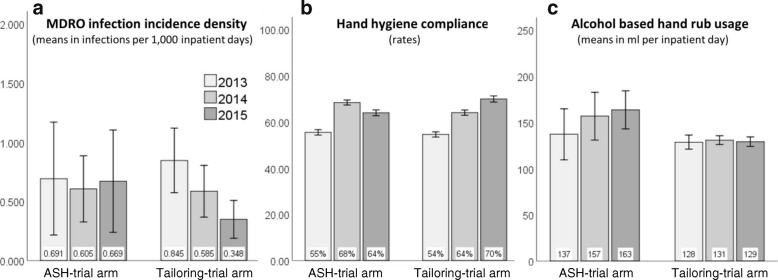
MDRO infection incidence density, hand hygiene compliance, and alcohol based hand rub usage during the study period of the PSYGIENE-C-RCT. Notes: Six wards per trial arm as per cluster-randomization. Error bars show +/− 1 standard errors, as appropriate. For results of significance of differences between trial arms within each year of the study period, see Table 2. For significance of differences across the study period within and across trial arms, see Table 3. Data on hand hygiene compliance in part b of the figure are from [15] and reprinted with permission

As Fig. 1(a) shows, incidence densities in the “ASH”-trial arm decreased in 2014 and increased again in 2015. MDRO-infection rates in the “Tailoring”-arm consistently decreased. While the difference in incidence rates between study arms was insignificant in every year (see Table 2(a)), the decrease from 2013 to 2015 in the “Tailoring”-arm by 0.497 infections per 1000 inpatient days was significant (*p* = 0.015; see Table 3(a)). The *p*-value for the interaction of this contrast (2015 vs. 2013) with the “trial arm”-factor (Tailoring vs. ASH) was 0.076.

**Table 2:** Tests for significance of differences between trial arms in MDRO infection incidence density (MDRO), hand hygiene compliance, and alcohol-based hand rub usage (AHRU) within each year of the study period, respectively (hand hygiene compliance data are from [15] and reprinted with permission)

	Tailoring-trial arm vs. ASH-trial arm in		
a) MDRO	Difference	95%-CI	*p*-value
2013	+0.154 i. per 1 k inpatient days	[-1.069; 1.376]	*p* = 0.785
2014	-0.020 i. per 1 k inpatient days	[-0.811; 0.771]	*p* = 0.956
2015	-0.322 i. per 1 k inpatient days	[-1.347; 0.704]	*p* = 0.501
b) Hand hygiene compliance	Difference	95%-CI	*p*-value
2013	-1%	[-4.1; 2.3]	*p* = 0.581
2014	-4%	[-7.5; -1.3]	*p* = 0.006
2015	+6%	[2.4; 9.5]	*p* = 0.001
c) AHRU	Difference	95%-CI	*p*-value
2013	-9 ml per inpatient day	[-72; 55]	*p* = 0.773
2014	-26 ml per inpatient day	[-84; 32]	*p* = 0.346
2015	-34 ml per inpatient day	[-82; 13]	*p* = 0.137

**Table 3:** Tests for significance of differences across the study period in MDRO infection incidence density (MDRO), hand hygiene compliance, and alcohol-based hand rub usage (AHRU), respectively, within and across trial arms (hand hygiene compliance data are from [15] and reprinted with permission)

	ASH-trial arm			Tailoring-trial arm			Comparison of trial arms	
a) MDRO	Difference	95%-CI	*p*-value	Difference	95%-CI	*p*-value	*F*	*p*-value
2014 vs. 2013	-0.086 i. per 1 k inpatient days	[-0.629; 0.456]	*p* = 0.730	-0.260 i. per 1 k inpatient days	[-0.803; 0.282]	*p* = 0.310	0.255	*p* = 0.625
2015 vs. 2014	0.064 i. per 1 k inpatient days	[-0.355; 0.483]	*p* = 0.739	-0.237 i. per 1 k inpatient days	[-0.656; 0.182]	*p* = 0.236	1.285	*p* = 0.283
2015 vs. 2013	-0.022 i. per 1 k inpatient days	[-0.400; 0.356]	*p* = 0.899	-0.497 i. per 1 k inpatient days	[-0.857; -0.119]	*p* = 0.015	3.927	*p* = 0.076
b) Hand hygiene compliance	Difference	95%-CI	*p*-value	Difference	95%-CI	*p*-value	*Chi* ^2^	p-value
2014 vs. 2013	+13%	[9.8; 16.1]	*p* < 0.001	+10%	[6.3; 12.6]	*p* < 0.001	2.3	*p* = 0.126
2015 vs. 2014	-4%	[-7.7; -1.2]	*p* = 0.007	+6%	[2.5; 9.3]	*p* = 0.001	18.5	*p* < 0.001
2015 vs. 2013	+9%	[5.1; 11.8]	*p* < 0.001	+16%	[11.9; 18.9]	*p* < 0.001	7.9	*p* = 0.005
c) AHRU	Difference	95%-CI	*p*-value	Difference	95%-CI	*p*-value	*F*	*p*-value
2014 vs. 2013	+20 ml per inpatient day	[-12.2; 51.4]	*p* = 0.199	+3 ml per inpatient day	[-29.7; 34.0]	*p* = 0.883	0.8	*p* = 0.407
2015 vs. 2014	+6 ml per inpatient day	[-10.2; 23.7]	*p* = 0.397	-2 ml per inpatient day	[-18.7; 15.2]	*p* = 0.827	0.6	*p* = 0.452
2015 vs. 2013	+26 ml per inpatient day	[-1.1; 53.8]	*p* = 0.058	+1 ml per inpatient day	[-27.0; 27.9]	*p* = 0.972	2.2	*p* = 0.168

### Effects on hand hygiene compliance and AHRU

Figure 1(b-c) shows that hand hygiene compliance but not AHRU inversely corresponded to these MDRO-infection rates. In the “Tailoring”-arm, compliance was higher in 2014 and 2015 than in the preceding year, respectively, while in the “ASH”-arm it decreased in 2015 after increasing in 2014. Most importantly, the increase in compliance between 2013 and 2015 was significantly larger in the “Tailoring”- than in the “ASH”-arm (*p* = 0.005; see Table 2(b)), and thus in line with the aforementioned difference between the trial arms in MDRO-infection reductions from 2013 to 2015. Baseline compliance rate in 2013 did not significantly differ between study arms in 2013, but both in 2014 and 2015 (*p* = 0.581, *p* = .006, and *p* = 0.001, respectively; see Table 3(b)). In contrast, AHRU did not correspond to infection rates: it remained almost stable in the “Tailoring”-arm, and the *p*-value for the increase in the “ASH”-arm from 2013 to 2015 was 0.058 (see Tables 2(c) and 3(c)).

## Discussion

To our knowledge, the PSYGIENE-study is the first C-RCT testing interventions which applied a psychological framework of behaviour change to healthcare workers’ hand hygiene compliance, and also assessed NIs. Specifically, on six experimental wards it found a significant decrease in MDRO infection incidence rates after two years following educational training sessions and feedback discussions which had been tailored on the basis of the HAPA as compliance model (“Tailoring”-study arm). In contrast, such a significant reduction was not found in six control wards which had received standard, i.e. untailored campaign interventions (“ASH”-arm). The difference between the decreases in the “Tailoring”- vs. the “ASH”-arm was not statistically significant (*p* = 0.076), to which complexities of interaction effects in field studies, and previously noted difficulties to detect them [49, 50], may have contributed. Thus, at most it can be viewed to represent an “interesting hint” [51], cf.49, p. 3. Besides increasing sample sizes, further research may try to avoid contamination, i.e. control groups receiving the intervention, by blinding staff who implement tailored educational and/or feedback interventions as wards’ allocations to study arms [52]. At the same time, the decrease in MDRO infection incidence rates in the “Tailoring”-arm corresponded to the increase in hand hygiene compliance from 2013 to 2015 in these wards, which was significantly higher than in the “ASH”-arm, and resulted in a significantly higher compliance rate in 2015 [15]. Such corresponding patterns were not found for AHRU.

Regarding the stronger improvement of hand hygiene compliance in the study arm in which educational training sessions and feedback discussions were tailored on the basis of the HAPA (i.e., + 16% in the in the second follow-up year, vs. + 9% in the control arm), this study is in line with the latest Cochrane review on tailored interventions to address determinants of practice [32]. This review, which had identified 32 studies which had dealt with professional behaviours of healthcare providers and included group-level interventions, had found that the odds of compliant practice were 1.56 higher after tailored vs. non-tailored interventions. Also, since the tailored interventions used in the PSYGIENE-trial (like the interventions of the above-mentioned HELPING HANDS-trial [7–9]) were explicitly grounded in a compliance theory from behavioural sciences – here, the HAPA –, explanations of its effects are possible on theoretical grounds as well. Given that practically all physicians and nurses who worked on the participating wards had reported before the trial to be motivated to comply with hand hygiene guidelines [17], the effect of the tailored interventions probably rested more on the interventions’ impacts on factors which promote the translation of motivation into compliance, i.e. action and coping planning, maintenance and relapse self-efficacy, and action control, and not so much on risk perceptions, outcomes expectancies and task self-efficacy (see Additional file 1: Table S1). Also, perceptions of the social environment were targeted, for instance by disseminating results of the questionnaire survey on low perceived support by team leaders and ward managers for hand hygiene compliance on one’s ward. In sum, on the level of psychosocial determinants of compliance, factors were addressed which take into account that hand hygiene not only represents a behaviour compromised by poor motivation, but a response prone to the intention-behaviour-gap, which may be bridged by internal and external cues to action.

Another result is noteworthy as well: only the study arm-specific developments in observed hand hygiene compliance across the study period, and not those regarding AHRU, inversely corresponded to the respective MDRO-infection rates. This may be seen as evidence that at least regarding NIs as outcomes, hand hygiene assessed by direct observation may a more valid measure of compliance than AHRU. This may be due to the fact that almost 6% of hand disinfections are unneeded, as a corollary study to the PSYGIENE-trial has shown on two of the trials ICUS [53]. While these unneeded disinfections should count as noncompliance (not least to save time, which is a limited resource in German healthcare [53]), they are included in AHRU. Interestingly, AHRU practically remained unchanged on wards having received tailored interventions.

### Limitations

Several limitations should be noted. First, while this study’s focus on MDRO-infections was deliberate, pooling MRGN-, VRE- and MRSA-infections was a less-than-ideal option chosen solely due to small number of cases. However, descriptive analyses revealed that incidence densities of MRGN-, VRE- and MRSA-infections in the “Tailoring”-arm decreased annually, while in the “ASH”-arm MRGN- and VRE-infection incidence densities increased from 2014 to 2015.

Second, while similar baseline levels of hand hygiene compliance pertained to the two study arms, this was not the case for ward size: while there were a total of 23,449 patient days in the “ASH”-arm in 2013 (2014: 20,934, 2015: 20,856), 38,111 patient days pertained to the “Tailoring”-arm at baseline (2014: 38,175, 2015: 37,814). Also, while the mean age of adult patients were more or less equivalent, mortality rates tended to be somewhat higher in the “Tailoring”-arm. Yet, the number of patient days remained stable across the three study years in the “Tailoring”-arm, and the percentage differences in mortality between the study arms remained relatively constant.

Third, while the decrease by nearly 0.5 infections per 1000 inpatient days in the “Tailoring”-arm is not trivial, it raises the issue of linearity of the association between compliance and infection rates. While this reduction corresponded to a 16%-increase in compliance, the compliance increase of 9% in the control arm was only matched by a reduction of 0.022 infections per 1000 inpatient days. This does not suggest linearity of the association, and further analyses of the associations between compliance levels and NI-rates are needed.

Fourth, it has to be stressed that the MDRO infection rate did not significantly differ between the study arms, i.e. in none of the three observed years. Thus, the trial achieved a significant reduction of MDRO-infections in the “Tailoring”-arm from 2013 to 2015, not a significantly lower rate than in the “ASH”-arm in 2015.

Fifth, the PSYGIENE-trial is a single-centre study on ICUs and hematopoietic stem cell transplantation units within one tertiary care university hospital. Generalizability to normal wards and general and secondary-level hospitals has to be tested.

Sixth, initiatives targeting MDROs other than the hand hygiene-related educational training sessions and feedback discussions were not modelled in this analysis. This also pertains to all other components of the local ASH-campaign. While there is no indication that these differed between study arms, it cannot be ruled out that the effects of the psychological tailoring were conditional on these other interventions. However, effects of two other potential influences can be ruled out: an antibiotic stewardship team was introduced at Hannover Medical School only after the trial, and daily antiseptic body washings with octenidine started only at the very end of the trial (late 2015) on only two ICUs.

Seventh, compliance observers were unblinded. Though they were affiliated to the Hospital Epidemiology Department, and thus not unit-based [54], this creates potential for observer biases, probably in favour of the “ASH”-arm due to a tendency of some staff members to feel committed to the campaign. This would work against the trial’s hypothesis. Also, while observation biases such as the Hawthorne effect have to be considered, there is no indication that such effects differed between the study arms.

Finally, interventions were restricted to educational training sessions and feedback discussions. One review reported that functions mostly aimed at in hand hygiene interventions on ICUs were education and training, persuasion, enablement, and environmental restructuring [55]. This reflects the notion of “standard multimodal programmes”, which have been assessed to overrate hand hygiene as a deliberative behaviour, and undervalue its habitual aspects [4]. While the PSYGIENE-trial did address this by considering habit-oriented constructs in tailoring, it does resemble “standard multimodal programmes” in its implementation strategies, which may explain why compliance rates of 80% or more were not attained.

### Suggestions for future research

Besides scrutinizing, as noted above, questions of the linearity of the association between hand hygiene compliance and NI incidences, and the generalizability of the present results to normal wards and general and secondary-level hospitals, future studies may try to combine the PSYGIENE-approach of psychological tailoring [15] with the team and leaders-directed HELPING HANDS-strategy [7–9]. That is, given that the HAPA-model presumes that factors such as social support strongly influence sustained behaviour change as well, this may contribute to promoting professional hand hygiene as a behavioural pattern of self-regulatory capacity and ongoing (self-)monitoring of compliance with guidelines within social environments which are conducive to infection prevention and patient safety. After all, one of the seminal reviews on interventions to promote hand hygiene in hospitals showed that the addition of goal setting and reward incentives, i.e. psychological measures, and accountability strategies, i.e. socio-environmental approaches, to the multimodal WHO-campaign can lead to further improvements [11]. In other words, different behavioural sciences should cooperate to overcome the abovementioned “standard multimodal programmes” [4], and take into account that “hand hygiene is best understood in terms of socio-cultural, organizational, perceptual, cognitive, and psychological determinants” [4], p. 208–9. Thus, strategies that target compliance determinants both at policy, organizational, leadership, team, and individual levels [6] may probably be able to achieve and sustain compliance improvements and NI reductions which exceed those in the HELPING HANDS- and the PSYGIENE-trial.

## Conclusion

This further analysis of data from the C-RCT PSYGIENE [15] provides evidence that psychologically tailored interventions may not only lead to more sustainable increases in hand hygiene compliance, but also – while not to a lower level of MDRO-infections – to a significant decrease in MDRO-infections on ICUs and hematopoietic stem cell transplantation units not found for untailored interventions. This effect of the tailored interventions was probably driven by habit-oriented factors such as action and coping planning, maintenance and relapse self-efficacy, action control, and perceptions of the social environment. As a corollary, results show that compliance increases do not imply increases in AHRU, as indicated by stability of AHRU across study years in the “Tailoring”-study arm. Further research should examine social-psychological and environmental extensions of psychologically tailored interventions, and their cost-effectiveness, to improve healthcare workers’ hand hygiene compliance, and ultimately to prevent NIs.

## Additional files

Additional file 1: **Table S1.** Compliance determinants according to the Health Action Process Approach (HAPA). (PDF 60 kb)

Additional file 2: **Table S2.** Behavior change techniques used in the PSYGIENE-trial. (PDF 74 kb)

Additional file 3: **Table S3.** Distribution of the 92 nosocomial MDRO-infections in 86 patients by pathogen. (PDF 104 kb)

## Abbreviations

AHRU: Alcohol-based hand rub usage
ASH: Aktion Saubere Hände (= German adaptation of WHO’s “Clean Care is Safer Care”)
C-RCT: cluster-randomized controlled trial
HAPA: Health Action Process Approach
ICU: intensive care unit
MDRO: multidrug-resistant organisms
MRGN: multidrug-resistant gram-negative bacteria
MRSA: methicillin-resistant *Staphylococcus aureus*
NI: nosocomial infection
PSYGIENE: PSYchological optimized hand hyGIENE promotion (= acronym of the trial’s title)
VRE: Vancomycin-resistant *Enterococcus*
WHO: World Health Organization
