# Supplementary material for: Impact of psychologically tailored hand hygiene interventions on nosocomial infections with multidrug-resistant organisms: results of the cluster-randomized controlled trial PSYGIENE
Source: Antimicrob Resist Infect Control. 2019 Mar 25;8:56. doi: 10.1186/s13756-019-0507-5 (PMC6434638; doi:10.1186/s13756-019-0507-5)
Supplement: Supplementary file 1 — Table S1. Compliance determinants according to the Health Action Process Approach (HAPA). (PDF 60 kb) [file 13756_2019_507_MOESM1_ESM.pdf]

Table S1: Compliance determinants according to the Health Action Process Approach (HAPA)

Construct	Definition	Background	Questionnaire item examples
**Risk perceptions**	Subjective assessments/ estimations of the probability and severity of negative events.	If the probability of nosocomial infections were exactly 0 and perceived as such, prevention measures would be unnecessary. A similar situation would hold if their severity were comparable to that of, e.g., a simple influenza infection. Thus, one psychological prerequisite for hand hygiene compliance is risk perception, which is multiplicatively composed of the subjective probability and severity of the negative event in question (i.e., in this context, the transmission of, colonization with, or infection with an MDRO).	How do you rate the probability that you will transfer pathogens …<br>- … given you do not disinfect your hands?<br>- … despite disinfecting your hands?
**Outcome expectancies**	Subjectively perceived associations between behaviours and certain preventive or other outcomes.	Given risk, people generally seek preventive measures. If people are subjectively convinced that transmissions, colonisations and infections can be avoided by hand hygiene compliance, these beliefs represent preventive outcome expectancies or response efficacy. However, hand hygiene can also be associated with consequences other than preventive effects. An employee may be convinced that he/she will be seen as a role model or receive recognition if he/she compliantly performs hand hygiene, but also that he/she will have time problems. Hand hygiene therefore has both benefits and costs. If expectations regarding potential consequences of future hand hygiene behaviours are generated based on such experiences, non-infection-related outcome expectancies develop that influence hand hygiene behaviour besides response efficacy.	- If I disinfect my hands before and after every potentially infectious task, I contribute to preventing infections.<br>- If I disinfect my hands before and after every potentially infectious task, I get skin problems on my hands.<br>- If I disinfect my hands before and after every potentially infectious task, I am a role model for my colleagues.
**Self-efficacy expectancies**	Beliefs that one has the ability or competence to perform one (or more) behaviour(s) using one's own or self-organized resources.	In addition to realistic risk perceptions and a positive cost–benefit ratio based on outcomes expectancies, self-efficacy expectancies are important in developing strong motivation to execute any given behaviour. E.g., even if an employee is convinced that hand hygiene is important to prevention and that it has more pros than cons, he/she will invest less motivational energy if he/she does not think he/she can successfully execute the behaviour (task self-efficacy). Similar assertions pertain to the maintenance of the behaviour and to recovery after relapse. Thus, self-efficacy beliefs of one's own ability to execute the behaviour are essential since for most people the motivation to achieve goals through own action and resources even in unfavourable circumstances and potential barriers is imperative.	I am confident to be capable of disinfecting my hands before and after every potentially infectious task even if…<br>- … this is not always easy to do.<br>- … this takes some time.<br>- … I had recently forgotten to do so.
**Goals intentions**	Intents to behave in certain ways not yet including planning, i.e. expressing the level of motivation to execute a particular behaviour.	Goal intentions are the outcome of motivational processes in terms of intents not yet including planning. One problem arises since people usually want to achieve many goals at any one time. This is also true of healthcare professionals. Thus it is even more important that the intention to comply with hand hygiene guidelines is strong (strong motivation). However, even then intentions are only moderate predictors of behaviour, metaphorically comparable to New Year's resolutions. It is therefore important to turn goals into actions. Psychologically, this step is modulated primarily by planning processes and action control.	- How strongly do you intend to disinfect your hands before and after every potentially infectious task?
**Implementation intentions (action and coping planning)**	Specific when/where/how plans related to behaviour (action planning) and how to deal with potential relapses (coping planning).	One problem of hand hygiene is its integration specific workflows. Plans may be promoted using problem-solving approaches, for example, in which factors that influence compliance are analysed and strategies to overcome barriers or to promote supportive factors are developed. In addition, detailed plans may be facilitated through practical advice. These plans should contain information on the context and on the frequency, duration, and intensity of the behaviour in question.	Of late, I have precisely planned …<br>- … how to deal with barriers and events impeding hand disinfection.<br>- … how I react after noticing that I forgot to disinfect my hands.
**Action control**	Awareness of behavioural standards, self-monitoring, and subjectively perceived self-regulatory effort.	In addition to implementation intentions and self-efficacy expectancies, action control plays a key role as a further self-regulatory strategy in behaviour execution. Its components are knowledge of the behaviours with which one complies with guidelines, self-monitoring, and subjectively perceived self-regulatory effort in relation to one's own behaviour. Action control can be promoted using techniques such as demonstration of the behaviour, behavioural rehearsal/practice, professional feedback on behaviour, and instruction on how to perform a behaviour. These techniques induce immediate availability and thus preserve mental resources.	- I know how to disinfect my hands according to the guidelines.<br>- I make sure that I disinfect my hands before and after every potentially infectious task.<br>- I have to make an effort to disinfect my hands before and after every potentially infectious task.
**External barriers and resources**	Perceived and actual fea-tures of the environment.	Any behaviour is a function of both the person and the environment. The perception of environmental factors such as, e.g., resources on one's ward and one's work environment more generally, involves beliefs about this environment. These in turn could be either barriers or incentives for compliance. For example, there may be too little infrastructure regarding patient safety or too little educational input on hygiene and infection prevention issues, and employees may believe that there is not enough support available to them to comply sustainably with recommended measures. These beliefs could subsequently inhibit intentions to comply. Environmental factors such as the infection control culture in a hospital may interfere with positive influences of knowledge on the amount of self-regulation needed to enact hand hygiene.	- Personnel resources in terms of physicians / nurses are appropriate on my ward.<br>- Resources regarding medical devices / space (e.g. for patient isolation) are appropriate.<br>- On my ward, one frequently has to deal with problems due to occupancy / absenteeism.<br>- On my ward, cooperation with colleagues / superiors / patients' relatives works well.
