# Supplementary material for: Impact of psychologically tailored hand hygiene interventions on nosocomial infections with multidrug-resistant organisms: results of the cluster-randomized controlled trial PSYGIENE
Source: Antimicrob Resist Infect Control. 2019 Mar 25;8:56. doi: 10.1186/s13756-019-0507-5 (PMC6434638; doi:10.1186/s13756-019-0507-5)
Supplement: Supplementary file 2 — Table S2. Behavior change techniques used in the PSYGIENE-trial. (PDF 74 kb) [file 13756_2019_507_MOESM2_ESM.pdf]

Table S2: Behavior change techniques used in the PSYGIENE-trial*

Behaviour change techniques used in both study arms	Behaviour change techniques used in the "Tailoring"-arm only
1.1 Goal setting (behaviour), e.g. visualization of the WHO-"Five moments for hand hygiene"	1.2 Problem solving, e.g. feedback discussions on optimizing sequences of workflow as assessed in problem- centred interviews
2.2 Feedback on behaviour, e.g. reflection of wards' compliance rates and respective rankings	1.4 Action planning, e.g. reflect and discuss implementation intentions as assessed in survey
2.4 Self-monitoring of outcome(s) of behaviour, e.g. presentation of compliance rates and discussion of options for monitoring on wards	3.2 Social support (practical), e.g. feedback discussions on optimizing dispenser provisions
2.7 Feedback on outcome(s) of behaviour, e.g. feedback on hospital-wide NI rates	3.3 Social support (emotional), e.g. active listening in feedback discussions to evoke reflection on balancing benefits and costs
3.1 Social support (unspecified), e.g. identification and forwarding of employees' ideas for improvement	4.4 Behavioural experiments, e.g. Fluorescence behaviour training by fluorescence methods using ultraviolet light boxes
4.1 Instruction on how to perform the behaviour, e.g. visualizations using task-related images	6.3 Information about others' approval, e.g. reflection of perceived recognition by superiors for compliance as assessed in survey
5.1 Information about health consequences, e.g. knowledge transfer on consequences of non-compliance and NIs	7.1 Prompts/cues, e.g. laminated information sheets distributed in wards' common rooms
5.3 Information about social and environmental consequences, e.g. knowledge transfer on economic consequences of NIs	8.1 Behavioural practice/rehearsal, e.g. exercises on hand hygiene in line with guidelines regarding glove use
5.6 Information about emotional consequences, e.g. knowledge transfer on psychological consequences of NIs	8.7 Graded tasks, e.g. focusing on individual indications such as before aseptic procedures
6.1 Demonstration of the behaviour, e.g. presentation of behaviour with fluorescence methods using ultraviolet light boxes	10.4 Social reward, e.g. awarding incentives to wards with highest compliance at university celebrations
6.2 Social comparison, e.g. comparison with compliance rates of other wards (blinded) in order to activate social behavioural norms	12.5 Adding objects to the environment, e.g. optimizing hygiene facilities to reduce self-regulatory effort
8.6 Generalisation of target behaviour, e.g. transfer of problem-solving approaches across indications	13.1 Identification of self as role model, e.g. illustration and discussion of the function of role models in hand hygiene compliance
9.2 Pros and cons, e.g. discussing effects of compliance and noncompliance	13.2 Framing/Reframing, e.g. raising the issue of compliance as a team task (team cooperation)
15.1 Verbal persuasion about capability, e.g. discussion of positive compliance development	14.6 Situation-specific reward, e.g. certification of ward with highest compliance with the trial
15.3 Focus on past success, e.g. discussion of best year	

* Labels for behaviour change techniques come from the taxonomy developed by Michie and colleagues[33].
