# Supplementary material for: Impact of psychologically tailored hand hygiene interventions on nosocomial infections with multidrug-resistant organisms: results of the cluster-randomized controlled trial PSYGIENE
Source: Antimicrob Resist Infect Control. 2019 Mar 25;8:56. doi: 10.1186/s13756-019-0507-5 (PMC6434638; doi:10.1186/s13756-019-0507-5)
Supplement: Supplementary file 3 — Table S3. Distribution of the 92 nosocomial MDRO-infections in 86 patients by pathogen. (PDF 104 kb) [file 13756_2019_507_MOESM3_ESM.pdf]

Table S3: Distribution of the 92 nosocomial MDRO-infections in 86 patients by pathogen*

		2013	2014	2015	TOTAL
**„Tailoring"-study arm**	MRSA	4	4	1	**9**
	VRE *E. faecium*	14	11	8	**33**
	MRGN	12	6	4	**22**
	**TOTAL**	**30**	**21**	**13**	**64**
	3MRGN *E. coli*	5	3	1	**9**
	3MRGN *P. aeruginosa*	1	1	1	**3**
	3MRGN *E. cloacae*	1	0	0	**1**
	3MRGN *K. pneumoniae*	1	2	0	**3**
	3MRGN *Acinetobacter*	1	0	0	**1**
	3MRGN other	0	0	0	**0**
	4MRGN *P. aeruginosa*	2	0	1	**3**
	4MRGN *K. pneumoniae*	0	0	0	**0**
	4MRGN *Acinetobacter*	1	0	0	**1**
	4MRGN *Citrobacter*	0	0	1	**1**
**„ASH"-study arm**	MRSA	0	3	1	**4**
	VRE *E. faecium*	0	2	4	**6**
	MRGN	9	5	4	**18**
	**TOTAL**	**9**	**10**	**9**	**28**
	3MRGN *E. coli*	3	2	0	**5**
	3MRGN *P. aeruginosa*	2	1	3	**6**
	3MRGN *E. cloacae*	1	0	1	**2**
	3MRGN *K. pneumoniae*	0	0	0	**0**
	3MRGN *Acinetobacter*	1	0	0	**1**
	3MRGN other	0	1	0	**1**
	4MRGN *P. aeruginosa*	2	0	0	**2**
	4MRGN *K. pneumoniae*	0	1	0	**1**
	4MRGN *Acinetobacter*	0	0	0	**0**
	4MRGN *Citrobacter*	0	0	0	**0**
**Total**	MRSA	4	7	2	**13**
	VRE *E. faecium*	14	13	12	**39**
	MRGN	21	11	8	**40**
	**TOTAL**	**39**	**31**	**22**	**92**
	3MRGN *E. coli*	8	5	1	**14**
	3MRGN *P. aeruginosa*	3	2	4	**9**
	3MRGN *E. cloacae*	2	0	1	**3**
	3MRGN *K. pneumoniae*	1	2	0	**3**
	3MRGN *Acinetobacter*	2	0	0	**2**
	3MRGN other	0	1	0	**1**
	4MRGN *P. aeruginosa*	4	0	1	**5**
	4MRGN *K. pneumoniae*	0	1	0	**1**
	4MRGN *Acinetobacter*	1	0	0	**1**
	4MRGN *Citrobacter*	0	0	1	**1**

*The terms 3MRGN vs. 4MRGN refer to a classification of Gram-negative MDROs in terms of their susceptibility against four classes of antimicrobials (acylureidopenicillins, third- and fourth-generation cephalosporins, quinolones, and carbapenems): a multi-resistant strain is classified as 3MRGN if resistant to three out of four classes, or as 4MRGN if resistant to all four classes.[48]
